# Supplementary material for: A critical role of action-related functional networks in Gilles de la Tourette syndrome
Source: Nat Commun. 2024 Dec 16;15:10687. doi: 10.1038/s41467-024-55242-6 (PMC11649905; doi:10.1038/s41467-024-55242-6)
Supplement: Supplementary file 1 — Supplementary Information [file 41467_2024_55242_MOESM1_ESM.pdf]

Supplement

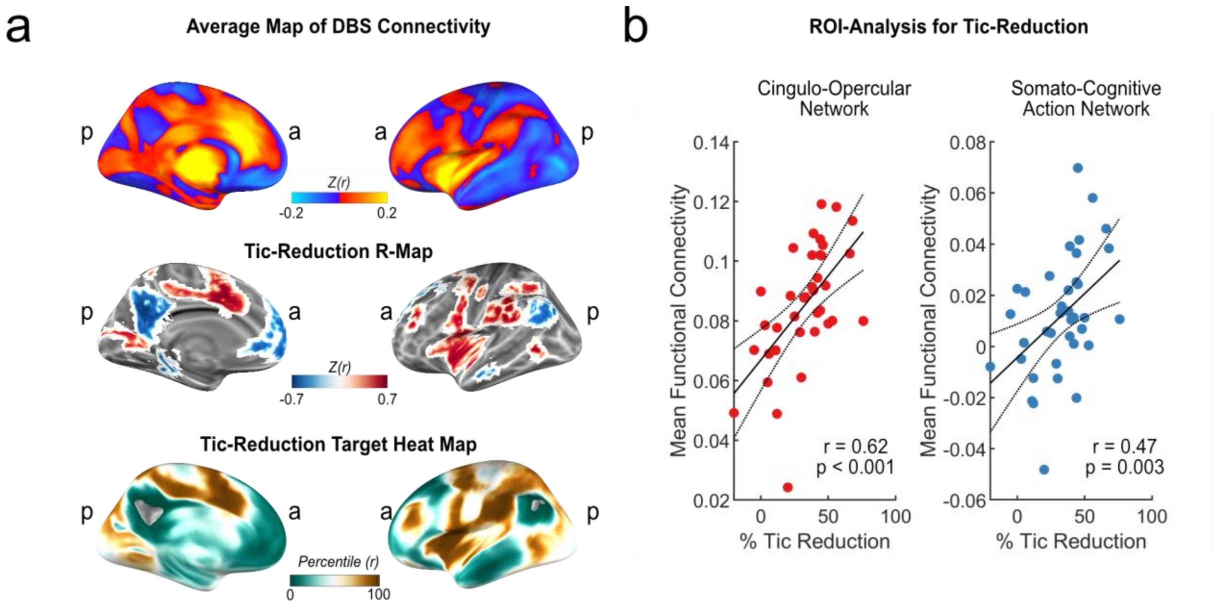

Supplemental Figure 1: Main Results Derived from Connectivity Maps of Electric Fields. *Using the electric fields as a weighted seed for calculating the individual stimulation maps instead of the binarized stimulation site resulted in highly similar results both on the whole brain level (a) and regarding the Region-of-Interest (ROI)-Analysis (b). Permutation-based, two-tailed spearman rank correlations showed significant correlations between tic reduction and connectivity to both the cingulo-opercular (r = 0.62; p = 0.0002) and somato-cognitive action network (r = 0.47; p = 0.003) (N = 37). All brain surfaces are shown on the left hemisphere. All scatter plots include linear trend lines with 95% confidence bounds. a = anterior; p = posterior.*

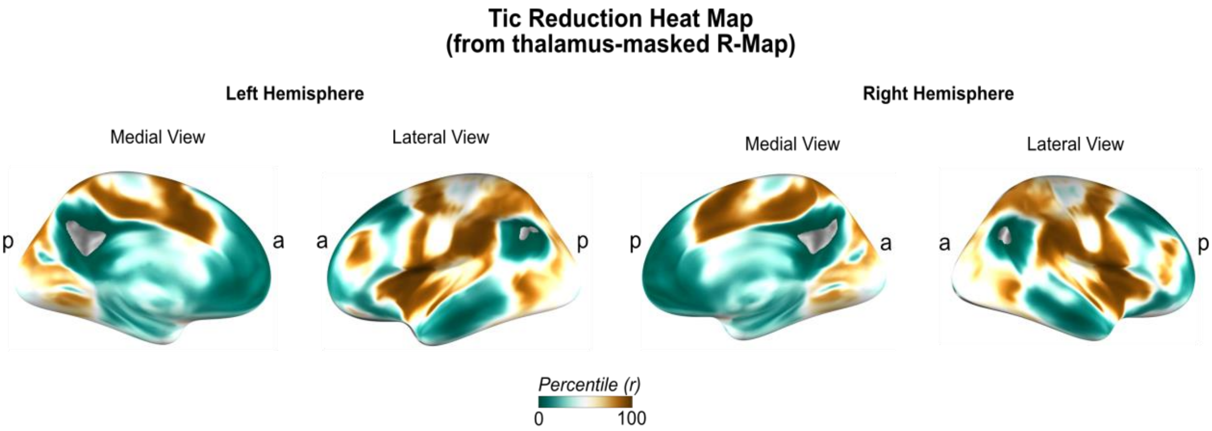

Supplemental Figure 2: Tic Reduction Heat Map, derived from thalamus-masked R-Map. *Displayed is the Heat Map that results from the Tic-Reduction R-Map, when only the thalamus is masked instead of the whole subcortex (see Figure 4). Both maps are highly similar, since the original R-Map included rarely any voxels in the subcortex that resided outside the thalamus.*

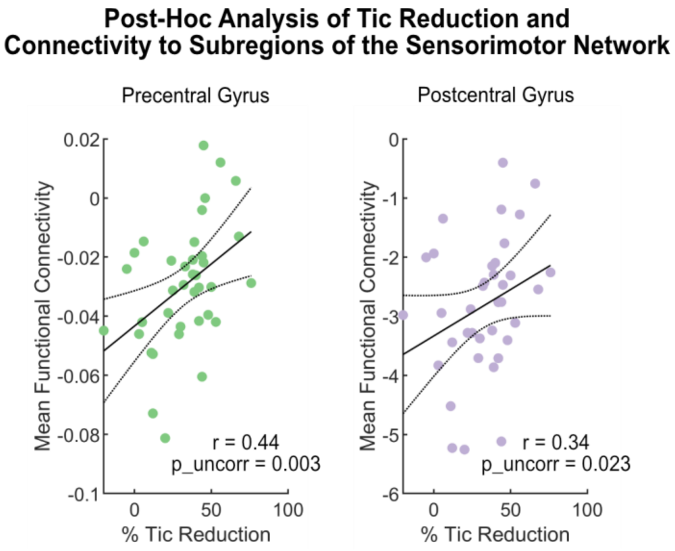

Supplemental Figure 3: Post-Hoc Analysis of Tic Reduction and Connectivity to Subregions of the Sensorimotor Network (SMN). *Since the SMN showed a trend towards significance when correlating its connectivity to stimulation sites with tic reduction, we analyzed which anatomical regions within the SMN contributed to this effect. In the Permutation-based, two-tailed spearman rank correlations, noth the precentral gyrus (r = 0.44, p_uncorrected = 0.003) and postcentral gyrus (r = 0.34, p_uncorrected = 0.023) demonstrated positive associations between connectivity and tic reduction (N = 37). However, in contrast to connectivity with the CON and SCAN, 92% of patients exhibited anticorrelation with the precentral gyrus, and 100% showed anticorrelation with the postcentral gyrus. This suggests a different mechanism compared to the connectivity patterns observed with the CON and SCAN. All scatter plots include linear trend lines with 95% confidence bounds. p_uncorr = uncorrected p-values*

Supplemental Table 1: Peak Values of Target Heat Map

REGION	HEMISPHERE	MNI COORDINATE			SPEARMAN *R*	VOLUME
		X	Y	Z		
**SUPRAMARGINAL GYRUS**	R	61	-27	31	0.77	3258
**INSULA/OPERCULUM**	R	45	10	2	0.64	4030
**SUPPLEMENTARY MOTOR AREA**	R	2	7	50	0.72	1979
**SUPRAMARGINAL GYRUS**	L	-61	-32	27	0.65	930
**INSULA/OPERCULUM**	L	-57	6	3	0.64	181

L = Left; R = Right. Standard coordinates in MNI space and Spearman's rank correlation coefficient of the peak voxel are reported. Cluster volumes are reported in mm³.

Supplemental Table 2

Participant	Preoperative Modality	Preoperative Voxel Size	Postoperative Modality	Postoperative Voxel Size
1	MRI	[0.5 0.5 1]	CT	[0.6 0.6 0.8]
2	MRI	[0.5 0.5 1]	CT	[0.5 0.5 0.8]
3	MRI	[0.5 0.5 1]	CT	[0.6 0.6 0.5]
4	MRI	[0.5 0.5 1]	CT	[0.5 0.5 0.8]
5	MRI	[0.5 0.5 1]	CT	[0.5 0.5 0.8]
6	MRI	[0.6 0.6 2]	CT	[0.5 0.5 2.8]
7	MRI	[0.5 0.5 1]	CT	[0.5 0.5 0.8]
8	MRI	[0.6 0.6 2]	CT	[0.6 0.6 0.8]
9	MRI	[0.6 0.6 2]	CT	[0.5 0.5 3]
10	MRI	[0.5 0.5 1]	CT	[0.6 0.6 0.8]
11	MRI	[0.5 0.5 1]	CT	[0.6 0.6 0.8]
12	MRI	[0.5 0.5 1]	CT	[0.5 0.5 0.5]
13	MRI	[0.6 0.6 2]	CT	[0.4 0.4 0.8]
14	MRI	[0.9 0.9 1]	CT	[0.7 0.7 3]
15	MRI	[0.5 0.5 1]	CT	[0.6 0.6 0.8]
16	MRI	[0.7 0.7 0.7]	CT	[0.6 0.6 0.8]
17	MRI	[0.5 0.5 1]	CT	[0.6 0.6 0.8]
18	MRI	[0.5 0.5 1]	CT	[0.4 0.4 0.6]
19	MRI	[0.5 0.5 1]	CT	[0.6 0.6 0.8]
20	MRI	[0.6 0.6 2]	CT	[0.4 0.4 5]
21	MRI	[0.6 0.6 2]	CT	[0.4 0.4 4.8]
22	MRI	[1 1 1]	MRI	[1 1 1]
23	MRI	[1 1 1]	MRI	[1 1 1]
24	MRI	[1 1 1]	MRI	[1 1 1]
25	MRI	[1 1 1]	MRI	[1 1 1]
26	MRI	[1 1 1]	CT	[0.6 0.6 3]
27	MRI	[1 1 1]	MRI	[1 1 1]
28	MRI	[0.5 0.5 0.5]	CT	[0.5 0.5 1.3]
29	MRI	[0.7 0.7 0.7]	CT	[0.5 0.5 1.3]
30	MRI	[0.7 0.7 0.7]	CT	[0.5 0.5 1.3]
31	MRI	[1 0.5 0.5]	CT	[0.5 0.5 1.3]
32	MRI	[0.5 0.5 1]	CT	[0.5 0.5 1.3]
33	MRI	[0.5 0.5 1]	CT	[0.5 0.5 1.3]
34	MRI	[0.5 0.5 1]	CT	[0.5 0.5 1.3]
35	MRI	[1 1 1]	CT	[0.5 0.5 1.3]
36	MRI	[0.5 0.5 1]	CT	[0.6 0.6 0.8]
37	MRI	[0.5 0.5 1]	CT	[0.6 0.6 0.8]

MRI = Magnetic Resonance Imaging. CT = Computer Tomography; voxel sizes reported in mm.
