# Supplementary material for: A critical role of action-related functional networks in Gilles de la Tourette syndrome
Source: Nat Commun. 2024 Dec 16;15:10687. doi: 10.1038/s41467-024-55242-6 (PMC11649905; doi:10.1038/s41467-024-55242-6)
Supplement: Supplementary file 2 — Reporting Summary [file 41467_2024_55242_MOESM2_ESM.pdf]

nature portfolio

Corresponding author(s): Juan Carlos Baldermann

Last updated by author(s): 08.11.2024

Reporting Summary

Nature Portfolio wishes to improve the reproducibility of the work that we publish. This form provides structure for consistency and transparency in reporting. For further information on Nature Portfolio policies, see our Editorial Policies and the Editorial Policy Checklist.

Statistics

For all statistical analyses, confirm that the following items are present in the figure legend, table legend, main text, or Methods section.

n/a	Confirmed	
☐	☒	The exact sample size (*n*) for each experimental group/condition, given as a discrete number and unit of measurement
☐	☒	A statement on whether measurements were taken from distinct samples or whether the same sample was measured repeatedly
☐	☒	The statistical test(s) used AND whether they are one- or two-sided<br>*Only common tests should be described solely by name; describe more complex techniques in the Methods section.*
☐	☒	A description of all covariates tested
☐	☒	A description of any assumptions or corrections, such as tests of normality and adjustment for multiple comparisons
☐	☒	A full description of the statistical parameters including central tendency (e.g. means) or other basic estimates (e.g. regression coefficient) AND variation (e.g. standard deviation) or associated estimates of uncertainty (e.g. confidence intervals)
☐	☒	For null hypothesis testing, the test statistic (e.g. *F*, *t*, *r*) with confidence intervals, effect sizes, degrees of freedom and *P* value noted<br>*Give P values as exact values whenever suitable.*
☒	☐	For Bayesian analysis, information on the choice of priors and Markov chain Monte Carlo settings
☒	☐	For hierarchical and complex designs, identification of the appropriate level for tests and full reporting of outcomes
☐	☒	Estimates of effect sizes (e.g. Cohen's *d*, Pearson's *r*), indicating how they were calculated

Our web collection on statistics for biologists contains articles on many of the points above.

Software and code

Policy information about availability of computer code

Data collection	Clinical Data were shared using Microsoft Excel
Data analysis	Electrode reconstructions and stimulation sites were processed using Lead-DBS software (Version 2.6, www.lead-dbs.org). Postoperative scans were aligned with preoperative MRI images using SPM 12 (https://www.fil.ion.ucl.ac.uk/spm/software/spm12/) for MRI and Advanced Normalization Tools (ANTs, http://stnava.github.io/ANTs/) for CT scans. A subcortical refinement step in Lead-DBS addressed potential brain shifts during surgery. Images were then normalized to the Montreal Neurological Institute (MNI) 2009b nonlinear asymmetric template space, utilizing the symmetric normalization method in ANTs and the subcortical refinement tool from Lead-DBS. Electrode positions were determined by the PaCER algorithm or the TRAC/CORE method within Lead-DBS. Electric fields were estimated based on individual stimulation settings using FastField within Lead-DBS. Stimulation<br>Functional connectivity maps and subsequent statistical analysis were performed in Lead-DBS v2.6 and using customized scripts within MATLAB R2022b (The MathWorks Inc., Natick, MA, USA).<br><br>All code used to preprocess the data including the estimation of individual connectivity maps is openly available within the Lead DBS software (https://github.com/leaddbs/leaddbs). Project-specific code to reproduce results and figures of this manuscript is openly available on OSF (DOI: 10.17605/OSF.IO/UPJVB).

For manuscripts utilizing custom algorithms or software that are central to the research but not yet described in published literature, software must be made available to editors and reviewers. We strongly encourage code deposition in a community repository (e.g. GitHub). See the Nature Portfolio guidelines for submitting code & software for further information.

Data

Policy information about availability of data

All manuscripts must include a data availability statement. This statement should provide the following information, where applicable:

- Accession codes, unique identifiers, or web links for publicly available datasets
- A description of any restrictions on data availability
- For clinical datasets or third party data, please ensure that the statement adheres to our policy

Source data for all figures, except those visualizing imaging findings, are provided with this paper in a Source Data file. The Source Data file includes all relevant raw data from scatter, bar, and box plots. The main group analysis imaging results are publicly available in the Open Science Framework (OSF) repository (DOI: 10.17605/OSF.IO/UPJVB). Individual patient imaging data cannot be openly shared due to privacy and data-sharing regulations. However, data can be requested under specific data use agreements from the primary investigators at each center. For such requests, the corresponding author (JCB) can be contacted to initiate the process of obtaining access in accordance with institutional and regulatory guidelines. The functional connectivity data used in this work is publicly available under https://dataverse.harvard.edu/dataset.xhtml?persistentId=doi:10.7910/DVN/25833.

Research involving human participants, their data, or biological material

Policy information about studies with human participants or human data. See also policy information about sex, gender (identity/presentation), and sexual orientation and race, ethnicity and racism.

Reporting on sex and gender	In the main analysis, we included 37 patients with severe GTS (5 females). Only the self-reported sex is given in the manuscript.
Reporting on race, ethnicity, or other socially relevant groupings	No description of race or ethinicity is available
Population characteristics	mean age: 32.4 years ± 10.8 standard deviations
Recruitment	Patients underwent DBS surgery according to clinical decision-making. We sourced data from adult patients with severe GTS undergoing DBS (N = 37) in three European centers: Cologne, Germany (n = 21); Milan, Italy (n = 8); and Hannover, Germany (n = 8). Additionally, the International Tourette Deep Brain Stimulation Database and Registry (henceforth, GTS-DBS-Registry) 24 provided data for further replication (N = 10). In the Cologne cohort, 12 of 21 patients participated in two prospective clinical trials25,26. From the first trial25, four patients lacked postoperative imaging adequately allowing electrode reconstruction in standard space, while from the second, one patient was excluded due to a suspected combination of GTS and functional tic-like behavior (FTLB) as previously described26. The Hannover cohort, derived from a further recent clinical trial, excluded two patients with GTS also suffering from comorbid FTLB as outlined in the original publication27. Milan's cohort was based on retrospective data of nine patients who received treatment following individual clinical decision-making. In this dataset, one patient had to be excluded due to insufficient postoperative imaging data. For external replication, we referenced previously published data from patients of the GTS-DBS-Registry who underwent thalamic DBS28. From this set, we excluded data whose source data was already used in this study or which was assessed outside the six to twelve months post-intervention, leaving ten patients.<br>There was no financial compensations for the subjects.
Ethics oversight	The study was carried out in accordance with the Declaration of Helsinki and approved by the local ethics committee at the University Hospital Cologne under approval number 23-1409-retro.

Note that full information on the approval of the study protocol must also be provided in the manuscript.

Field-specific reporting

Please select the one below that is the best fit for your research. If you are not sure, read the appropriate sections before making your selection.

☒ Life sciences ☐ Behavioural & social sciences ☐ Ecological, evolutionary & environmental sciences

For a reference copy of the document with all sections, see nature.com/documents/nr-reporting-summary-flat.pdf

Life sciences study design

All studies must disclose on these points even when the disclosure is negative.

Sample size	In the main analysis, we included 37 patients with severe GTS. Patients with GTS undergoing DBS are very rar. Here we colleted the largest sample size published so far. Considering an expected effect size of 0.45 according to preliminary analysis (Baldermann & Hennen et al., BioPsych CNNI 2022), a sample size of 33 would be suitable to reject the null-hypothesis that treatment outcomes depend on functional DBS connectivity (assuming a Power of 0.8 and an alpha error of 0.05.
Data exclusions	Three patients were excluded due to a suspected functional tic-like behaviour as described in the papers of the original data.
Replication	We used an independent dataset from the GTS-DBS-Registry that was independently prepocessed to replicate the main findings. Additionally, we employed tic-inducing lesions to test the role of action-related functional networks. In both approaches, the role of action-related

	functional networks was confirmed
Randomization	not applicable for the current analysis, since real-life clinical outcomes were used for analysis
Blinding	not applicable, since real-life clinical outcomes were used for analysis

Reporting for specific materials, systems and methods

We require information from authors about some types of materials, experimental systems and methods used in many studies. Here, indicate whether each material, system or method listed is relevant to your study. If you are not sure if a list item applies to your research, read the appropriate section before selecting a response.

Materials & experimental systems

n/a	Involved in the study
☒	☐ Antibodies
☒	☐ Eukaryotic cell lines
☒	☐ Palaeontology and archaeology
☒	☐ Animals and other organisms
☐	☒ Clinical data
☒	☐ Dual use research of concern
☒	☐ Plants

Methods

n/a	Involved in the study
☒	☐ ChIP-seq
☒	☐ Flow cytometry
☐	☒ MRI-based neuroimaging

Clinical data

Policy information about clinical studies

All manuscripts should comply with the ICMJEguidelines for publication of clinical research and a completedCONSORT checklist must be included with all submissions.

Clinical trial registration	Data were retrospectively collected, data from two previously published clinical trials were included (NCT03958617 and DOI: 10.1016/j.brs.2021.04.004) were included
Study protocol	Not applicable
Data collection	All participants with complete neuroimaging and clinical outcome scores in the respective primary assesment were included, as desciribed above, three participants were excluded due to suspected functional tic-like behaviour. . As follow up, the earliest assessment in a period between six to twelve months post-intervention was chosen.
Outcomes	We used the percentage change in the Yale Global Tic Severity Scale (YGTSS) global score (comprising the total tic score and impairment score) as the primary outcome parameter. In three patients from the Milan cohort, the impairment score was not available and thus only the total tic score was used. Of note, the global and total tic scores show a highly similar correlation with the clinically evaluated disease severity and thus serve as comparable outcome parameters.

Plants

Seed stocks	Not applicable
Novel plant genotypes	Not applicable
Authentication	Not applicable

Magnetic resonance imaging

Experimental design

Design type	Retrospective analysis of individual stimulation-dependent functional normative connectivity
Design specifications	not applicable
Behavioral performance measures	not applicable

Acquisition

Imaging type(s)	Structural MRI and CT for electrode reconstruction, fMRI from an openly available dataset of 1,000 healthy participants (Yeo et al. 2011, J Neurophysiol)
Field strength	3 or 1.5 Tesla for structural MRI; 3Tesla for fMRI
Sequence & imaging parameters	Preoperative T1 and T2-weithed MRI, postoperative T1-weighted MRI or CT; for fMRI, gradient-echo echo-planar imaging (EPI) sequences were obtained with the following parameters_ repetition time (TR) = 3,000 ms, echo time (TE) = 30 ms, flip angle (FA) = 85°, 3 × 3 × 3-mm voxels, field of view (FOV) = 216, and 47 axial slices collected with interleaved acquisition and no gap between slices
Area of acquisition	Whole Brian

Diffusion MRI ☐ Used ☒ Not used

Preprocessing

Preprocessing software	SPM 12, ANTs, Lead-DBS, Matlab
Normalization	ANTS, SPM 12
Normalization template	ICBM 2009b NLIN asymmetric non-linear 2009b MNI152
Noise and artifact removal	Biasfield correction
Volume censoring	none

Statistical modeling & inference

Model type and settings	Mass univariate analysis
Effect(s) tested	Spearman correlation between percentage symptome reduction and functional connectivity estimates

Specify type of analysis: ☐ Whole brain ☐ ROI-based ☒ Both

Anatomical location(s): Predefined action-related networks, i.e. cingulo-opercular network and somato-cognitive action network

Statistic type for inference (See Eklund et al. 2016)	Spearman correlation between voxel-wise and avraged ROI connectivity estimates
Correction	FDR-correction at pFDR <0.05

Models & analysis

n/a	Involved in the study
☐	☒ Functional and/or effective connectivity
☒	☐ Graph analysis
☒	☐ Multivariate modeling or predictive analysis

Functional and/or effective connectivity	For each bilateral pair of stimulation sites, the averaged functional connectivity across the 1,000 subjects to the rest of the brain was computed by sampling time series from voxels within the binary stimulation sites in each subject of the normative connectome and correlating these time series with those of every other voxel. This procedure resulted in a stimulation-dependent functional connectivity map for each patient, containing Fisher-z-transformed connectivity strengths that was used for subsequent group analysis
